# Exosomes and Signaling Nanovesicles from the Nanofiltration of Preconditioned Adipose Tissue with Skin-B^®^ in Tissue Regeneration and Antiaging: A Clinical Study and Case Report

**DOI:** 10.3390/medicina60040670

**Published:** 2024-04-21

**Authors:** Fabiano Svolacchia, Lorenzo Svolacchia, Patrizia Falabella, Carmen Scieuzo, Rosanna Salvia, Fabiana Giglio, Alessia Catalano, Carmela Saturnino, Pierpaolo Di Lascio, Giuseppe Guarro, Giusy Carmen Imbriani, Giuseppe Ferraro, Federica Giuzio

**Affiliations:** 1 Department of Sense Organs, University of Rome “La Sapienza”, 00184 Rome, Italy; 2 Department of General Surgery, University of Rome “La Sapienza”, 00184 Rome, Italy; lorenzo.svolacchia@hotmail.it; 3 Department of Sciences, University of Basilicata, 85100 Potenza, Italy; patrizia.falabella@unibas.it (P.F.); carmen.scieuzo@unibas.it (C.S.); r.salvia@unibas.it (R.S.); fabiana.giglio@unibas.it (F.G.); carmela.saturnino@unibas.it (C.S.); 4 Spinoff XFlies S.R.L, University of Basilicata, Via Dell’Ateneo Lucano 10, 85100 Potenza, Italy; 5 Department of Pharmacy-Drug Sciences, University of Bari “Aldo Moro”, 70126 Bari, Italy; alessia.catalano@uniba.it; 6 Department of General Surgery AOR San Carlo, Basilicata, 85100 Potenza, Italy; pierpaolo.dilascio@ospedalesancarlo.it; 7 Department of Plastic and Reconstructive Surgery, ASL Umbria 1, Umbria, 06127 Perugia, Italy; giuseppe.guarro@uslumbria1.it; 8 Department of Surgical Oncology, Aorn Sant’Anna e San Sebastiano, Campania, 81100 Caserta, Italy; carmen.imbriani@aorncaserta.it; 9 Department of Medicine and Health Sciences “Vincenzo Tiberio”, University of Molise, 86100 Campobasso, Italy; 10 Spinoff TNcKILLERS s.r.l., University of Basilicata, 85100 Potenza, Italy; federica.giuzio@unibas.it; 11 U.O.C. Primary Care and Territorial Health, Social and Health Department, State Hospital, 47893 San Marino, Italy

**Keywords:** tissue regeneration, nanovesicles, exosomes, micro RNA, Jaluexos

## Abstract

*Background and Objectives*: This three-year clinical trial aimed to demonstrate that only the signaling vesicles produced by ADSCa, containing mRNA, microRNA, growth factors (GFs), and bioactive peptides, provide an advantage over classical therapy with adipose disaggregate to make the tissue regeneration technique safer due to the absence of interfering materials and cells, while being extremely minimally invasive. The infiltration of disaggregated adipose nanofat, defined by the Tonnard method, for the regeneration of the dermis and epidermis during physiological or pathological aging continues to be successfully used for the presence of numerous adult stem cells in suspension (ADSCa). An improvement in this method is the exclusion of fibrous shots and cellular debris from the nanofat to avoid inflammatory phenomena by microfiltration. *Materials and Methods*: A small amount of adipose tissue was extracted after surface anesthesia and disaggregated according to the Tonnard method. An initial microfiltration at 20/40 microns was performed to remove fibrous shots and cellular debris. The microfiltration was stabilized with a sterile solution containing hyaluronic acid and immediately ultrafiltered to a final size of 0.20 microns to exclude the cellular component and hyaluronic acid chains of different molecular weights. The suspension was then injected into the dermis using a mesotherapy technique with microinjections. *Results*: This study found that it is possible to extract signaling microvesicles using a simple ultrafiltration system. The Berardesca Scale, Numeric Rating Scale (NRS), and Modified Vancouver Scale (MVS) showed that it is possible to obtain excellent results with this technique. The ultrafiltrate can validly be used in a therapy involving injection into target tissues affected by chronic and photoaging with excellent results. *Conclusions*: This retrospective clinical evaluation study allowed us to consider the results obtained with this method for the treatment of dermal wrinkles and facial tissue furrows as excellent. The method is safe and an innovative regenerative therapy as a powerful and viable alternative to skin regeneration therapies, antiaging therapies, and chronic inflammatory diseases because it lacks the inflammatory component produced by cellular debris and fibrous sprouts and because it can exclude the mesenchymal cellular component by reducing multiple inflammatory cytokine levels.

## 1. Introduction

Skin tissue harvested and processed at different sizes between 50 and 100 microns can be used effectively on scars due to the presence of tissue progenitors [1]. Adipose tissue can provide more viable tissue progenitors for micrografts between 50 and 100 microns through a simple procedure [2]. From 1 mL of lipoaspirate, approximately 2.0 to 6.0 × 10^7^ cells can be obtained with a cell viability of 90% [3], and, from one gram of the same tissue, 5 × 10^4^ progenitor cells can be isolated, although with debris in the suspension [4]. The uniqueness of adipose tissue is that it is possessed by every individual and is easily accessible for sampling, even with simple instruments such as a syringe and a needle of adequate size. The procedure for obtaining progenitors, which is the fraction of adult mesenchymal stem cells from adipose tissue without vital adipocytes, involves extraction and disaggregation according to the method of Tonnard et al. [5]. However, to obtain progenitor cells in a suspension without inflammatory components such as fibrous shots and cellular debris, which are responsible for the activation of the Toll-like (TL) system [4], the adipose tissue must be subjected to microfiltration after disaggregation [6,7]. The progenitor cells have to undergo cytofluorometry [8] to acquire the characteristics of adult stem cells. Through the phenomenon of plasticity, progenitor cells can induce the formation of new tissues via the formation of new cells that improve the characteristics of the receiving tissue [7].

The phases of tissue regeneration take place through a series of interactions between progenitors and newly formed cells immersed in the extracellular matrix, blood vessels, signals mediated by signaling proteins, and some regenerative microRNAs produced by the progenitors [9]. The method used in this study makes it possible to induce the same tissue regeneration mechanisms, with greater biological safety, using only the signaling of microvesicles produced by the progenitors, since these are the cells that transmit the signaling proteins [10]. These microvesicles, called exosomes, are capable of transmitting information to cells, having therapeutic effects through proteins and mRNAs and the microRNAs they contain [10].

Exosomes, by definition, are spherical or elliptical vesicles with a size varying between 50 and 200 nanometers (0.05–0.2 μm). They are mediators of all the cellular activities that produce them [10], which is achieved by releasing their information inside the target cells, as reported in the ARVO conference [11], with therapeutic activity on the cells of tissues different from them [12]. When exosomes are released from adult stem cells, they remain active even in the absence of the cells that produced them, activating the tissue regeneration process [10]. Exosomes can maintain the functional therapeutic phenotype of the adult stem cells that produced them by influencing tissue responses to lesions and positively interacting with cell metabolism [13].

When derived from healthy tissues, they induce risk-free regeneration [14], where bioactive lipids, nucleic acids, mRNAs, and microRNAs induce a regenerative biological response in the recipient cells. Exosomes can induce and activate biological functions even in senescent or dysfunctional cells, limiting the acquired expression of the senescent phenotype and preventing the expression of MMPs [15]. They can inactivate the TL4/NF-κB inflammatory cascade by reducing TLR4 levels [16]; they can reduce IFN-γ, TNF-α, and IL-1β levels, reducing local inflammatory phenomena [17]; and they can increase the expressions of TGF-β1 and IL-10 [18]. The exosomes derived from viable precursors of adipose micrografts induce a noninflammatory phenotype in macrophages toward the M2 phenotype [19], and they regulate autophagy [20]. In lesions of the dermis and epidermis, they allow more rapid physiological healing through the transfer of their microRNAs [21]. They promote endothelial formation, reduce oxidative stress damage, and improve nitric oxide synthesis [22]. The viable precursors of adipose tissue micrografts can release exosomes with the presence of microRNA-126. This microRNA can protect cells from acute events typical of hypoxia–reperfusion pathology by regulating neo-angiogenesis and endothelial cells [22,23]. They can restore the efficiency of the connection of the membrane potential of superoxide dismutase (SOD1) [24] and can inhibit elastase through the release of alpha-1-antitrypsin (AAT) [25] in the tissues, which results in a wide limitation of tissue aging phenomena. Exosomes induce plasticity in dermal fibroblasts [26] and allow physiological neo-collagenogenesis [27].

The exclusive use of exosomes makes it possible to design therapy that excludes the cells that have secreted them, thus allowing low immunogenicity [28]. Exosomes express the Alix protein on their surface [29], which is an adaptor protein associated with the cytoskeleton that regulates the function of tyrosine kinase (TK) and CD63, which play fundamental roles in cells by regulating development, activation, growth, and motility. Endothelial cell lines defined as HUVECs are activated by exosomes, suggesting that they promote angiogenesis in vitro and in vivo [30,31]. They reduce tissue degeneration by reducing apoptosis [32]. They improve the outcome of wounds and scars by increasing fibroblast proliferation and migration [33] and Wnt/β-catenin signaling [34] and by upregulating gene expression in the recipient tissues [35]. They allow over-regulation in the cells of proliferative markers such as cyclin D1, cyclin D2, cyclin A1, and cyclin A2; and growth factors such as VEGFA, PDGFA, EGF, and FGF2; and they stimulate and activate the AKT and ERK pathways, leading to a significant increase in re-epithelialization, physiological collagen deposition, and neovascularization in dermal lesions [36]. Adipose-derived adult stem cells are capable of producing a significant amount of exosomes [37], and this phenomenon occurs in both normoxic and hypoxic environments [33,38], with a positive functional impact on the receiving cells [38]. By using ADSCa-derived exosomes, it is possible to transfer a large amount of information into tissues [38], but ADSCa-derived exosomes must be separated from interfering components such as cellular debris and fibrous processes [39].

Exosomes from ADSCs can be obtained by extraction using filters of the appropriate size [40,41]. Specifically, exosomes derived from hypoxic ADSCs have a size that can vary from 20 to 300 nanometers (0.02–0.3 microns), with an average size of 90 nanometers (0.09 microns) [42]. ADSCa can be preconditioned without any manipulation to modulate the composition of the exosomes they secrete [43,44], from which the profiles of 148 microRNAs have been isolated [45]. Proteomic analysis has identified 1466 proteins that positively interfere with cellular functions [46]. The exosomes released by the previously conditioned adult stem cells allow a greater therapeutic effect [47], and preconditioning without manipulation is emerging as a key strategy to improve the functions of ADSCa to obtain exosomes that improve their efficacy in regenerative medicine [48,49]. This three-year clinical trial aimed to demonstrate that the signaling vesicles produced by ADSCa, containing mRNAs, microRNAs, GFs, and bioactive peptides in the phenomena of chrono- and photoaging of facial tissues, have an advantage over therapy with disaggregated adipose defined nanofat to make the tissue regeneration technique safer and minimally invasive. This study’s objective was to apply and evaluate the feasibility of a specific protocol rather than comparing control groups undergoing different procedures. This approach utilized established and validated methods to assess, through cytofluorometry, the presence, quality, and quantity of signaling vesicles released by adipose-derived adult stem cells.

## 2. Material and Methods

A total of 72 female patients aged between 34 and 68 years (mean age 48 years) were studied. They signed an informed consent for the use of lipoaspirate for the procedures described. This study was approved by the local ethics committee under protocol number 367/2021 and was conducted in accordance with the tenets of the Declaration of Helsinki. Skin-B^®^ 5 mL sterile solution containing amino acids and nonviscoelastic macromolecular hyaluronic acid was from Italfarmacia (Rome, Italy).

None of the patients had inflammatory pathologies of the dermis or epidermis, except for the presence of age-dependent phenomena and photoaging. No unapproved substances such as proprietary products or drugs were used in this study under conditions other than those approved. The presence of nanovescicles was determined by positive selection using an EV Isolation Kit Pan, Human of Milteniy Biotec Company, Bergisch Gladbach, North Rhine-Westphalia, Germany. The EV Isolation Kit Pan for humans facilitates the isolation of intact exosomes or extracellular vesicles (EVs) from cell culture supernatant, plasma, urine, or ascites. The isolation is performed by positive selection using MicroBeads recognizing the tetraspanin proteins CD9, CD63, and CD81. The isolation protocol is based on the renowned MACS technology, which enables fast isolation of high-purity and high-yield EV. The Visual Analogue Scale (VAS), NRS, and Berardesca Scale were used for data collection [50]. In addition, the MVS was used to document changes in scarring outcomes over time; in our study, it was used to assess the overall improvement in skin appearance, taking into account the three parameters mentioned above (stability, softness, and hydration).

## 3. Results

**Figure 1:**
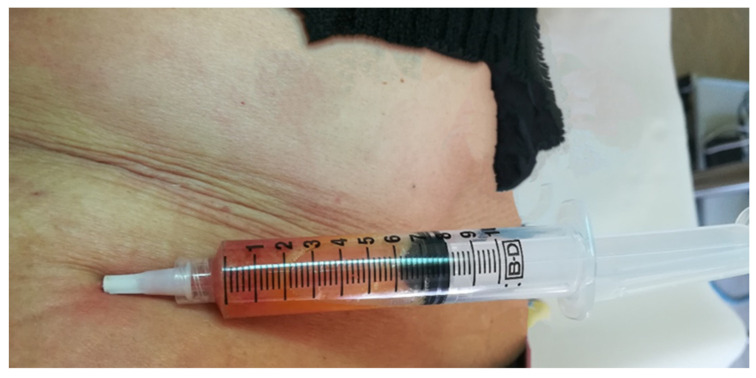
Extraction of adipose tissue.

After identification of the donor area, adipose tissue extraction and local infiltration with Klein’s solution were carried out as a method of anesthesia, and, after waiting for the whitening of the area induced by the presence of adrenaline in the solution contained therein, a total of 3.5 mL of adipose tissue was extracted using a 10 mL luer-lock syringe and a 16 G needle or with a multiport small-hole cannula (Figure 1). The tissue sample was decanted for 15 min to remove the anesthetic fluids, yielding 3 mL of adipose tissue, which was immediately disaggregated using the classic Tonnard method [5].

**Figure 2:**
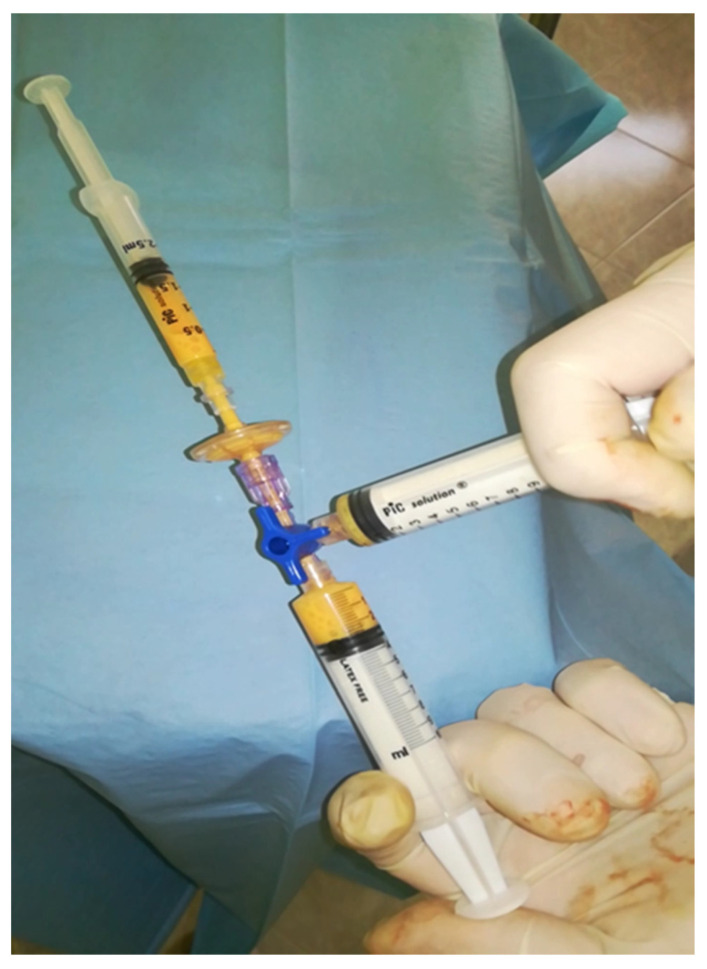
Fragmentation and simultaneous filtration at 20/40 microns.

During the disaggregation between the two syringes and employing a three-way tap, simultaneous filtration was carried out through a filter at 20/40 microns connected to one end (Figure 2). The filtration at 20/40 microns during the disaggregation of the tissues made it possible to eliminate the fibrous shots and the larger cellular debris protecting the side population in the harvesting syringe [5].

**Figure 3:**
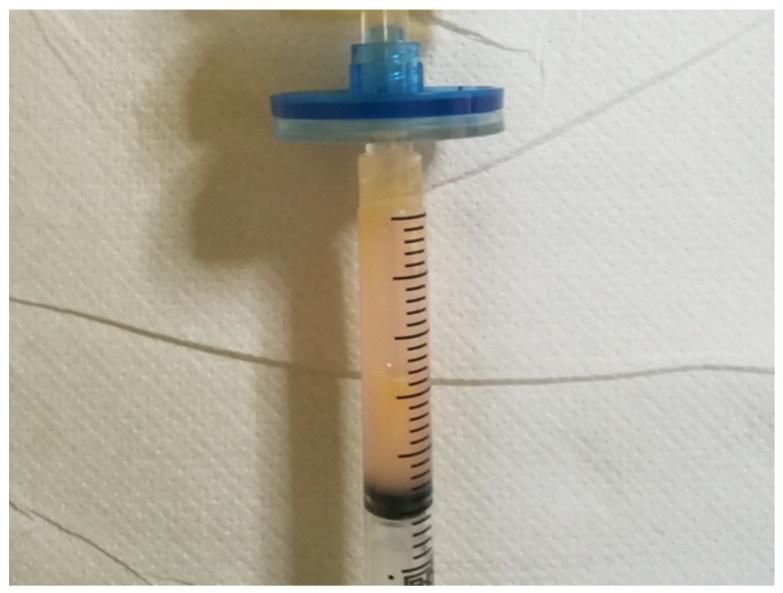
The 0.20-micron filtration.

A vial of Skin-B^®^ 5 mL sterile solution containing amino acids and nonviscoelastic macromolecular hyaluronic acid was added to the tissue thus obtained to condition the ADSCa and then was ultrafiltered to the final dimensions of 0.20 microns (200 nanometers) with an appropriately sized filter (Figure 3). The 200-nanometer ultrafiltration also guaranteed the exclusion of hyaluronic acid chains of different molecular weights from the final suspension to avoid influencing the clinical results of hyaluronic acid on the skin. The exosomes were isolated using only a size-based ultrafiltration technique [42,43].

**Figure 4:**
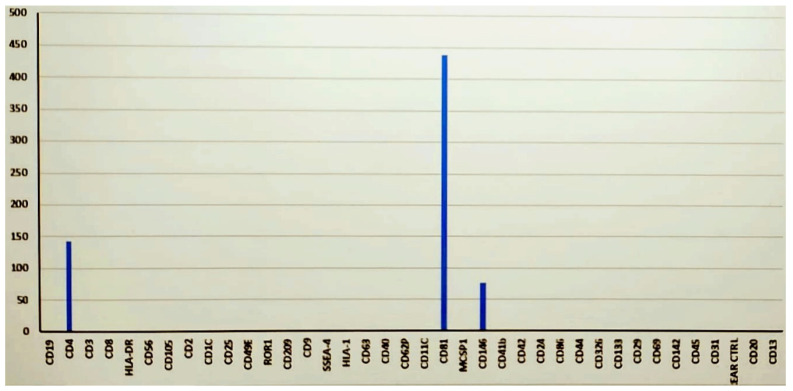
Flow cytometry of the suspension obtained after ultrafiltration.

This ultrafiltration technique made it possible to obtain microvesicles that did not contain almost any of the components of the interfering adipose disaggregate [27,38]. The large number of exosomes that may be lost during the ultrafiltration process is compensated for by an extremely fast, reproducible, painless, and minimally invasive technique since it involves the extraction of only 3.5 mL of adipose tissue, which can provide approximately 6.0 × 10^7^ cells with a cell viability of 90% [3,4]. Confirmation of the presence of exosomes in the microfiltrate was obtained by testing the procedure using an EV Isolation Kit, which allows the specific isolation of intact exosomes or EVs from cell culture supernatant, plasma, urine, or ascites. Isolation is performed by positive selection using microbeads that recognize tetraspanin proteins. The isolation protocol is based on MACS technology, which enables the rapid isolation of high-purity and high-yield EVs. Through this procedure, it was verified that, despite the final filtration procedure at 0.2 microns (200 nanometers), there were still particles covering part of the characterization signal, but we could see the presence of numerous vesicles using exosomal marker CD81, typical of regenerative functions [51], and the mesenchymal/endothelial marker CD146, specific for ADSCa after stabilization [52] (Figure 4).

**Figure 5:**
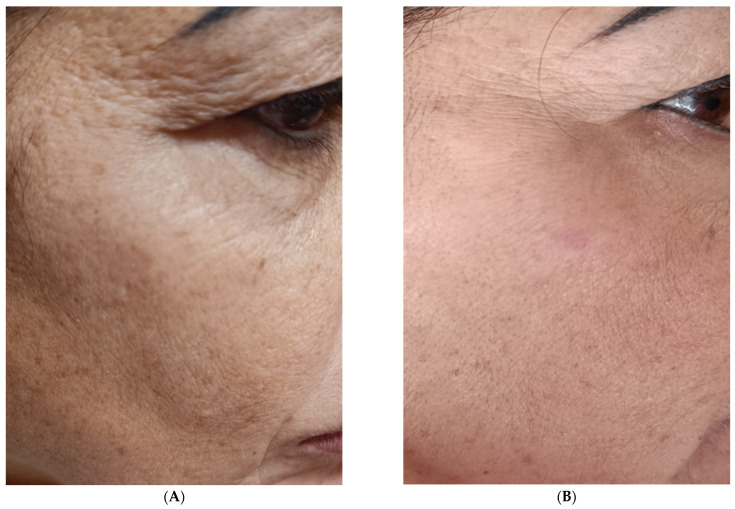
(**A**) Before the infiltration treatment; (**B**) 30 days after treatment.

However, the dilution of a fat disaggregate is always necessary because of the possible contraindications that a final filter of 0.20 microns may have for lipid emulsions. The final suspension containing the microinjections was, by convention, sterile since it was obtained at values around 0.2 microns. This procedure resulted in a final suspension of 3 mL (Figure 5). Once the suspension was obtained, it was injected with the same syringe and a 30 G 6 mm needle using the mesotherapy microinjection technique over the whole face, with the needle inclined at 45°, releasing a minimum amount of suspension when the needle was withdrawn until the formation of a visible wheal. However, this is not considered a drug, so we did not know the dosage or quantification. Additionally, it was not possible to know a priori the number of exosomes produced by the adult mesenchymal cells contained in each adipose tissue sample before treatment. We can state that, in the standardized sampling from each patient and examined by flow cytometry, we highlighted 450 million secretory vesicles with the CD81 marker. The procedure lasted about 30/40 min for each patient.

After the first clinical results on the use of 0.20-micron ultrafiltration [41] using only a physiological saline solution as mechanical support for the 0.2-micron filters, additional patients were enrolled and subjected to a clinical study and using an even higher-purity ultrafiltrate with a solution containing a vial of Skin-B^®^ 5 mL sterile solution containing amino acids and nonviscoelastic macromolecular hyaluronic acid. This procedure was called Jaluexos, both as filter support and as preconditioning, to modulate the composition of microvesicles produced by ADSCa [43,44] and the CD44 expressed on them to have a greater number of mRNAs and microRNAs to address regenerative simulation using a solution of Dulbecco’s modified Eagle medium [8].

A significant improvement in skin parameters was observed using this method. Compared to D0 (pretreatment), at a follow-up of 15 and 30 days after a single treatment, patients assessed their satisfaction with the treatment received by assigning scores for fine lines and wrinkles using a scale of 0 to 4 for each criterion (0 = unsatisfactory; 4 = satisfactory), as suggested by Berardesca et al. [53]. In addition, the individual signs of wrinkles and defect severity obtained for each treatment and each area were objectively assessed using a 10–0 NRS with separate scores for each area (10 = signs of high wrinkle severity or signs of high defect severity; 5 = signs of medium wrinkle severity or average defect severity; 0 = signs of low wrinkle severity or average defect severity) This scale provided a numerical measure of the severity of a general facial defect and, more specifically, the severity of wrinkles before the start of treatment (D0) (Figure 5A) and during follow-up (D30 in Figure 5B).

**Figure 6:**
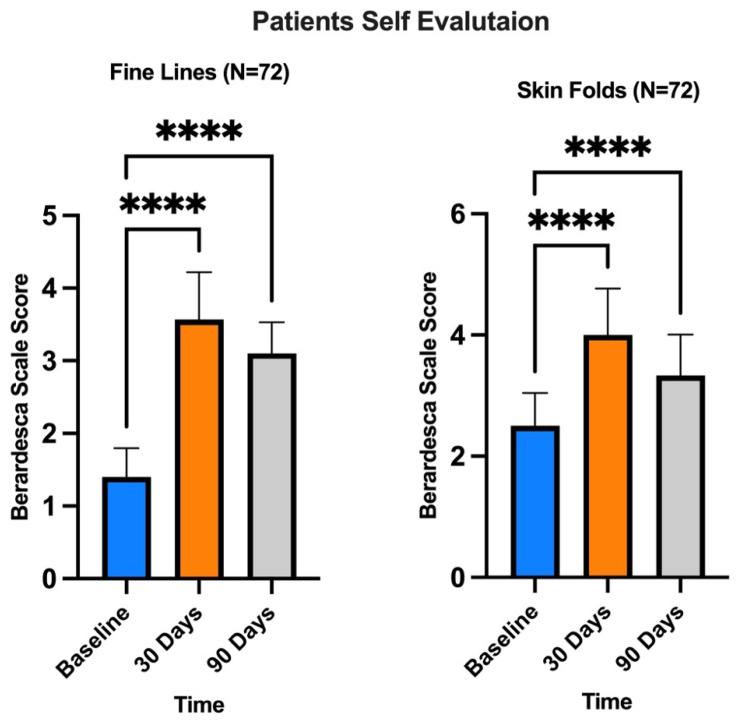
Berardesca Scale for patient satisfaction evaluation. Subjects evaluated their satisfaction in comparison to D0 (before treatment), 30 days and 90 days after treatment, by giving scores on firmness and cutaneous relief. Scale of 0–4 for each criterion (0 = unsatisfactory; 4 satisfactory). **** *p* < 0.0001 (one-way ANOVA).

**Figure 7:**
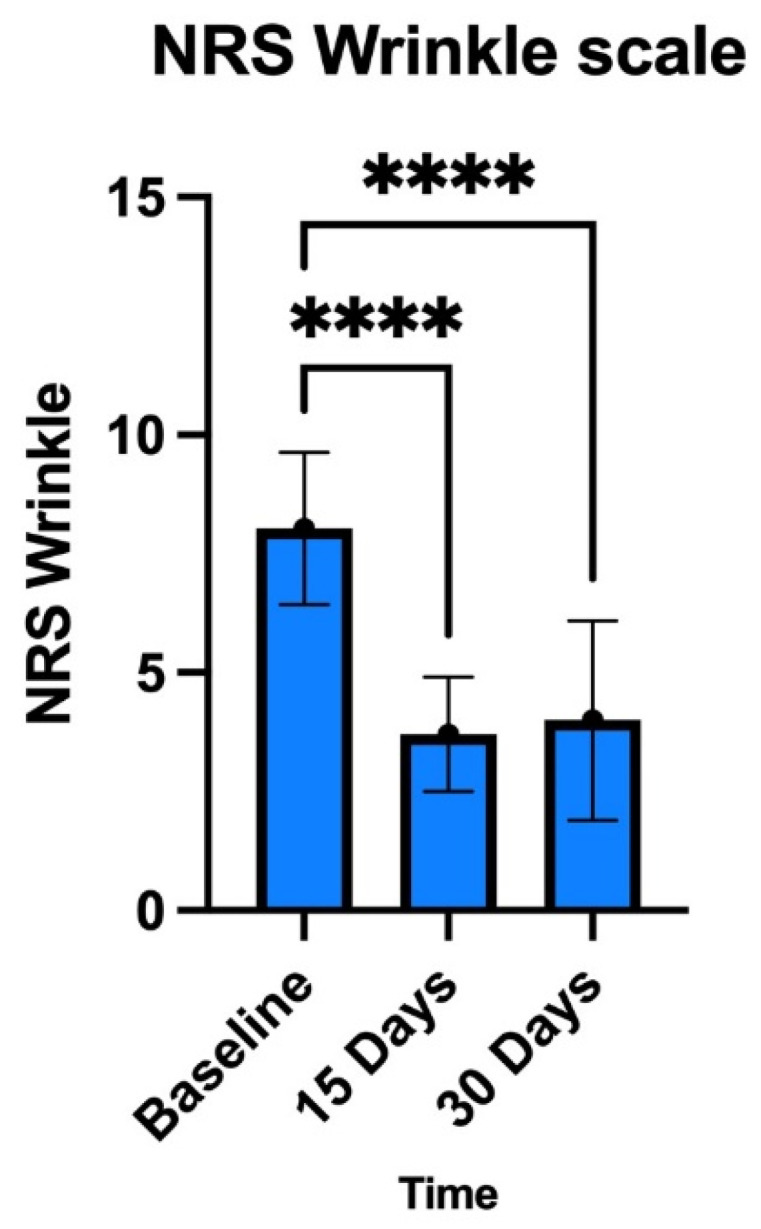
Numeric Rating Scale (NRS) evaluating defect severity and wrinkles: 10–0 scale with separate scores for each site (10 = wrinkle or defect severity; 5 = medium wrinkle signs or medium defect severity; 0 = low wrinkle signs or medium defect severity); *p* < 0.05. **** *p* < 0.0001.

The results presented in Figure 6 and Figure 7 show that treatment with exosomes induced a reduction in the signs of tissue aging in all patients.

**Figure 8:**
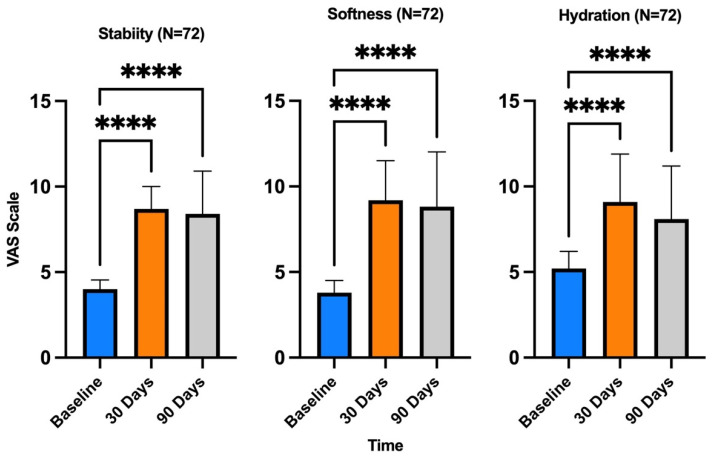
Modified Vancouver Scale used for the evaluation of stability, softness, and hydration. The Modified Vancouver Scale was used to estimate the improvement in skin appearance 15 days and 30 days after treatment in comparison to D0 (before treatment). The parameters considered were stability, softness, and hydration. **** *p* < 0.0001 (one-way ANOVA).

The MVS was also used for follow-up evaluation, and the parameters analyzed were stability, softness, and hydration, as shown in Figure 8. The treatment was found to be extremely safe with the method used, and no adverse effects were recorded.

## 4. Discussion

It was hypothesized that ADSCa-derived signaling vesicles and exosomes could be extracted from adipose tissue disaggregated according to the method described by Tonnard et al. [5], microfiltered at 20/40 microns, conditioned with Skin-B^®^, and nanofiltrated. The stages of tissue regeneration take place through a series of interactions between newly formed cells immersed in the extracellular matrix, blood vessels, signals mediated by signaling proteins, and the microRNAs produced by them. It was hypothesized that tissue-regeneration mechanisms could be induced using only signaling microvesicles produced by tissue progenitors with greater biological safety and that microvesicles would be able to transfer information employing proteins, mRNAs, and the microRNAs contained in them to cells, having a therapeutic effect. We aimed to clinically demonstrate that there was an improvement in the skin and to verify the presence of exosomes in the 200-nanometer nanofiltrate by flow cytometry. We used the Berardesca Scale, NRS and VAS. All three scales are valid, reliable, and appropriate for use in clinical practice, although the VAS is more difficult to use than the others. For general purposes, the NRS has good sensitivity and generates data that can be analyzed for various purposes. The exosomes from ADSCs can be obtained by extraction using filters of the appropriate size, and those derived from normoxic ADSCs and those derived from hypoxic ADSCs have a size that can vary from 20 to 300 nanometers (0.02–0.3 microns), with an average size of 90 nanometers (0.09 microns). ADSCs can be preconditioned without any manipulation to modulate the composition of the exosomes they secrete. Preconditioning facilitates the hypoxia of ADSCa, and the secretome of hypoxia-preconditioned ADSCa plays an important role in promoting cell proliferation and migration, improving angiogenesis, and inhibiting apoptosis and inflammation. The exosomes released by the previously conditioned adult stem cells produce a greater therapeutic effect, and preconditioning without manipulation is emerging as a key strategy for improving the functions of ADSCa to obtain exosomes with improved efficacy in regenerative medicine. All patients were satisfied with the treatment. The physical examination that the patients underwent during the follow-up was in line with their self-assessment.

## 5. Conclusions

It is now known that cell-based communication, even at a distance, occurs through signaling microvesicles, defined as exosomes. There are no other clinical studies on skin chrono- and photoaging using signaling microvesicles obtained by extraction from adipose tissue using a simple nanofiltration technique from preconditioned ADSCa, diluted in suspension but without any manipulation. Stabilization by the binding of hyaluronic acid to the CD44 of ADSCa positively affects the quality and number of microvesicles in the suspension. This clinical study showed that it is possible to extract signaling microvesicles with the typical markers CD81 and CD146 using a simple ultrafiltration system. The extraction of exosomes by ultrafiltration through a 0.20-micron filter eliminated the cellular components as well as sterilized the solution [54]. The method proved to be safe and fits into the field of innovative regenerative therapies as a powerful and viable alternative to epidermal regeneration therapies.

## Data Availability

The stored data is currently covered by privacy.

## Abbreviations

AAT	Alpha-1-antitrypsin
ADSCa	Adult mesenchymal stem cells (MSCa) derived from adipose tissue
EGF	Epidermal growth factor
EVs	Extracellular vesicles
FGF2	Fibroblast growth factor
GF	Growth factor
IFN-γ	Interferon-γ
IL-1β	Interleukin-1β
IL-1017	Interleukin-1017
MMPs	Matrix metalloproteinases
NF-κB	Nuclear factor kappa B
NRS	Numeric Rating Scale
PDGFA	Platelet-derived growth factor A
SOD1	Superoxide dismutase
TGF-β1	Transforming growth factor β1
TK	Tyrosine kinase
TLR4	Toll-like receptor 4
TNF-α	Tumor necrosis factor-α
VAS	Visual Analogue Scale
VEGFA	Vascular endothelial growth factor A
